# Enhancing the nutritional quality and digestibility of citronella waste (*Cymbopogon nardus*) for ruminant feed through ammoniation and fermentation techniques

**DOI:** 10.14202/vetworld.2024.1603-1610

**Published:** 2024-07-26

**Authors:** Dicky Pamungkas, Iman Hernaman, Mizu Istianto, Budi Ayuningsih, Simon Petrus Ginting, Solehudin Solehudin, Paulus Cornelius Paat, Mariyono Mariyono, Gresy Eva Tresia, Rina Ariyanti, Fitriawaty Fitriawaty, Yenni Yusriani

**Affiliations:** 1 Research Center for Animal Husbandry, Research Organization for Agriculture and Food, National Research and Innovation Agency of The Republic of Indonesia, Bogor, Indonesia; 2 Department of Animal Nutrition and Feed Technology, Faculty of Animal Husbandry, Padjadjaran University, Sumedang, Indonesia; 3 Research Center for Horticultural and Estate Crops, Research Organization for Agriculture and Food, National Research and Innovation Agency of The Republic of Indonesia, Bogor, Indonesia

**Keywords:** ammoniation, *Cymbopogon nardus*, digestibility, fermentation, *Trichoderma harzianum*

## Abstract

**Background and Aim:**

Citronella grass (*Cymbopogon nardus*) waste, produced by distilling citronella to produce essential oil, has a high potential for use as animal feed. However, the presence of high lignin content could limit its digestibility, prompting the need for treatment to improve its quality. This study aimed to improve the nutritional value and *in vitro* digestibility of ammoniated and fermented citronella waste (CW).

**Materials and Methods:**

The treatments of CW included CW without treatment as a control (T0), ammoniation of CW with urea (T1), fermentation of CW with *Trichoderma harzianum* (T2), and a combination of ammoniation and fermentation (amofer) of CW (T3). This study employed a randomized block design with five replicates for each of the four treatments. If there was a significant effect (p < 0.05), a *post hoc* Duncan’s multiple range test was performed to analyze the variance of the data.

**Results:**

The process of ammoniation and fermentation led to a notable increase in crude protein (2%–6%) while decreasing crude fiber (2%–6%), neutral detergent fiber (NDF) (5%–14%), acid detergent fiber (ADF) (5%–9%), lignin (4%–9%), and cellulose (2%–10%). The treatments enhanced the digestibility of dry matter, organic matter (OM), NH_3_, and total volatile fatty acid by 4%–12%, 6%–19%, 0.9–10 mM, and 35–142 mM, respectively. The decrease in NDF, ADF, acid detergent lignin (ADL), and cellulose fractions was accompanied by an improvement in dry matter and OM digestibility in CW. Ammoniated-fermented (amofer) CW, followed by fermentation with *T. harzianum* and ammoniated urea treatment, significantly enhanced the nutritional content and *in vitro* digestibility. The decrease in NDF, ADF, ADL, and cellulose fractions led to an improvement in dry matter and OM digestibility in CW.

**Conclusion:**

The application of amofer treatment with *T. harzianum* maximizes CW’s nutritional value and digestibility, making it the most efficient preservation method. Research is needed to explore the potential use of *Aspergillus* spp. and *Pleurotus* spp. for fermenting CW as ruminant fodder.

## Introduction

Citronella (*Cymbopogon nardus*) grass oil is an essential oil commodity with a significant prospect among other prominent essential oils and has a substantial share in the global market. Indonesia is the world’s 3^rd^ biggest supplier of citronella oil, with an estimated 40% market share [1, 2]. In 2014, the citronella oil-producing plantation area in Indonesia covered 19,300 hectares, yielding a plant productivity of 3,100 tons [3]. For every 100 g of citronella leaves distilled, only 0.03–1.53 g of essential oil are obtained, while the remaining 98.47–99.97 g of biomass are discarded as waste [4]. Citronella waste (CW) contains crude protein (CP) 7%−11.97%, crude fiber (CF) 25.14%−25.73%, ether extract (EE) 0.87%−2.35%, ash 7.91%−9.93%, nitrogen-free extract 45.2%, and total digestible nutrient (TDN) 50.76% [5, 6]. Elihasridas *et al*. [5] reported neutral detergent fiber (NDF), acid detergent fiber (ADF), neutral fiber carbohydrate, and lignin at 71.07%, 35.02%, 35.02%, and 2.8%, respectively. Sari *et al*. [7] revealed CP and CF contents as 5.82% and 35.02% of DM, respectively. The residue can be used in the daily basal diet of ruminants as an alternative source of fiber. The CW’s notable CF content poses a limitation. A suitable processing method enhances the fiber materials’ nutritional value and digestibility.

Nutritional value and digestibility of roughage can be improved by either ammoniation or fermentation [8−15], subsequently boosting ruminant performance [16−21]. Both rumen fermentation and digestibility improved *in vitro* [5, 22], as well as ruminant performance [23, 24], when fed ammoniated and fermented CW. Elihasridas *et al*. [5] found that a 25% incorporation of ammoniated and fermented *C. nardus* waste into a ruminant diet can replace *Pennisetum purpureum*. Twenty one days supplementation of 30% CW in rations for thin-tailed sheep did not decrease semen quality [23]. Using fermented CW for 14 days as a substitute for basal feed did not lower semen quality in thin-tailed sheep, according to Mariana *et al*. [24]. Although studies have been conducted on the influence of ammoniation and fermentation treatment, as well as their combination (ammoniation-fermentation/amofer), on CW’s chemical composition and digestibility, a considerable gap in understanding remains.

The potential of lignocellulosic fungi, such as Trichoderma, to release enzymes that break down cell walls [18] and enhance the nutritional value of CW is particularly promising. *Trichoderma* produces cellulolytic enzymes that significantly contribute to efficient silage fermentation [11]. The combined action of cellulolytic microorganisms and ammonifiers during fermentation enhances the nutritional value of roughage. The ammoniation process separates lignin and carbohydrate linkages within the cell wall structure, according to Oladosu *et al*. [25]. The expansion of the structural fibers on the particle surface creates an increased number of microbial attachment sites.

This study aimed to evaluate the nutritional value and *in vitro* digestibility of CW ammoniated and fermented with *Trichoderma* *harzianum* as a potential ruminant feed.

## Materials and Methods

### Ethical approval

Ethical approval for animal research was not required as live animals were not used in this study.

### Study period and location

The study was conducted from August to October 2022 at the Faculty of Animal Husbandry, Padjadjaran University, West Java, Indonesia.

### Diet preparation and treatment

CW was obtained from a privately owned citronella oil refinery in Sukabumi Regency, West Java, Indonesia. Using a chopper, CW was chopped into small pieces with a length of 3–5 cm and then sun-dried for 3−4 days until completely dry. The treatment for CW preservation consisted of a control (T0), ammoniation with urea (T1), fermentation with *T. harzianum* (T2), and a combination of ammoniation and fermentation (amofer) (T3).

500 g of CW was ammoniated by spraying it with a 4% urea solution, prepared by mixing urea and water in a 32:500 (w/v) ratio. Following a 21-day anaerobic incubation, ammoniated CW was gathered for analysis. 500 g of CW was incorporated into a plastic bag and coated evenly with a 90 mL liquid medium containing 6% *T. harzianum* biomass. 60 mL of squares was used to dissolve PDA containing 30 g of *T. harzianumin* to prepare the liquid medium. For 8 days, the CW was incubated under anaerobic conditions. 500 g of CW underwent a 21-day ammoniation process followed by an 8-day fermentation, both conducted under anaerobic conditions. CW was collected at specific intervals (T1: 21 days post-ammoniation, T2: 8 days post-fermentation, and T3: 29 days post-amofer) for nutritional content and *in vitro* digestibility assessment.

### Chemical analyses

The dry matter, ash, EE, CP, and CF content were determined according to the Association of Official Analytical Chemists (AOAC) [26] procedure for proximate analysis. The calcium and phosphorus content were measured through a duplicate method as specified by the AOAC [26]. Proportions of lignin, cellulose, NDF, and ADF within the sample were assessed as per methods described by Van Soest [27]. The total digestible nutrient content was calculated based on the methods described by Sutardi [28].

### *In vitro* procedure

*In vitro* digestibility was assessed as per the technique of Tilley and Terry [29]. 50 mL McDougall buffer, 25 mL rumen fluid (from cattle), and a 0.75-g sample were mixed in an *in vitro* tube. In the experiment, five tubes of each treatment were incubated for 48 h in a shaker water bath at 39°C. 48 h post-incubation, the tube contents were filtered using Whatman paper No. 41, dried at 105°C for 24 h to determine dry matter content, then ashed at 450°C–600°C for 8 h in an electric furnace to establish organic matter (OM) digestibility. Following the addition of blanks, the difference between the initial and residual weights of the samples was used to determine DM and OM content *in vitro*. The initial and residual matter was employed to calculate the *in vitro* digestibility of cellulose, ADF, and NDF. The supernatant was used to assess the ammonia (N-NH3) concentration [30] and total volatile fatty acid (TVFA) content using a steam distillation procedure [31].

### Statistical analysis

Data were tested for significant differences among treatments using analysis of variance and Duncan’s multiple range test, with a significance level of p < 0.05. The statistical analyses were carried out using SAS software 9.0 (SAS Institute Inc., Cary, NC, USA), while correlograms were created using R Studio 4.2.0 (https://posit.co/download/rstudio-desktop/).

## Results

Significant differences in the nutrient content (DM, ash, CP, CF, EE, Ca, and P) were observed among treatments, as shown in Table-1. A drop in the DM content (p < 0.0001) occurred in all treatment preservations (T1, T2, and T3), with the greatest loss of DM content observed in the ammoniation process (T1) reaching around 40%. Pretreatment of CW, on the other hand, significantly increased CP content (p < 0.0001) and total digestibility nutrient (p < 0.0001). Moreover, there was a significant decrease in CF and EE content (p < 0.0001) across all treatments. In addition, treating CW with ammonia and/or *T. harzianum* significantly declined (p < 0.0001) fiber fractions of ADF, NDF, acid detergent lignin (ADL), and cellulose. However, all treatments had no significant effect on the hemicellulose content (p < 0.2634). The results showed the treatments of CW significantly increased CP (2%−6%) and decreased CF (2%−6%), NDF (5%−14%), ADF (5%−9%), lignin (4%−9%), and cellulose (2%−10%).

**Table-1:** Nutritional profile of CW in various preservation treatments (%DM).

Chemical composition	Treatment				SEM	p-value

	T0	T1	T2	T3		
DM	91.38^a^	46.82^d^	73.98^b^	53.62^c^	4.04	<0.0001
OM^a^	91.40	92.33	91.8^b^	91.49	0.16	0.1675
Ash	8.60	7.67	8.15	8.51	0.16	0.1707
CP	7.72^d^	12.84^b^	10.05^c^	13.66^a^	0.55	<0.0001
CF	32.00^a^	29.74^b^	29.13^b^	25.39^c^	0.57	<0.0001
EE	2.56^a^	2.60^a^	1.51^c^	1.97^b^	0.12	<0.0001
NFE^b^	49.11^b^	47.15^c^	51.16^a^	50.46^ab^	0.42	0.0002
Ca	0.82^a^	0.62^ab^	0.35^a^	0.38^a^	0.07	0.0213
P	0.19^d^	0.81^c^	2.36^a^	1.87^b^	0.26	<0.0001
TDN	46.62^d^	51.75^c^	50.03^b^	55.59^a^	0.76	<0.0001
NDF	75.38^a^	71.42^b^	67.26^c^	64.77^d^	0.97	<0.0001
ADF	68.62^a^	63.46^b^	60.99^c^	58.82^d^	0.85	<0.0001
ADL	15.60^a^	11.29^b^	8.16^c^	6.11^d^	0.84	<0.0001
Cellulose	49.62^a^	46.66^b^	40.31^c^	39.15^c^	1.02	<0.0001
Hemicellulose	6.77	7.96	6.27	5.95	0.38	0.2634

^a,b,c,d^With divergent superscripts, the means within the similar row differ significantly (p < 0.05), T0=Control, T1=Ammoniated citronella waste, T2=Fermented citronella waste with *T. harzianum*, T3=Ammoniated-fermented (amofer) citronella waste, SEM=Standard error of mean, CW=Citronella waste, DM=Dry matter, OM=Organic matter, CP=Crude protein, CF=Crude fiber, EE=Ether extract, NFE=Nitrogen-free extract, Ca=Calcium, P=Phosphor, TDN=Total digestibility nutrient, NDF=Neutral detergent fiber, ADF=Acid detergent fiber, ADL=Acid detergent lignin.

a OM is based on the following formula=Crude protein + Ether extract + Crude fiber + Nitrogen free extract.

b NFE is based on the following formula=100 – Dry matter – Crude protein – Ether extract – Crude fiber

**Figure-1:**
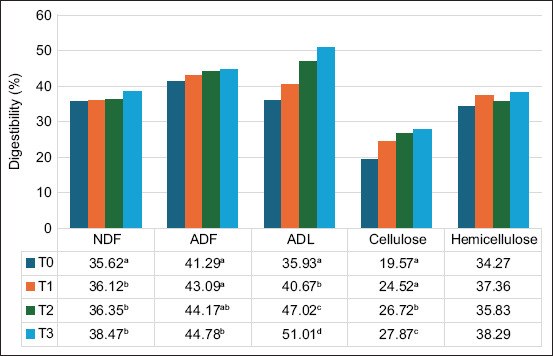
Fiber content digestibility of each treatment. T0=Control, T1=Ammoniated citronella waste, T2=Fermented citronella waste with *Trichoderma harzianum*, T3=Aammoniated-fermented (amofer) citronella waste, NDF=Neutral detergent fiber, ADF=Acid detergent fiber.

The digestibility of *in vitro* dry matter digestibility (IVDMD) and *in vitro* organic matter digestibility (IVOMD), TVFA, and NH_3_ concentration were significantly different among the treatments (Table-2). Our study also demonstrated that the process of ammoniation and/or fermentation had a significant increase (p < 0.0001) in IVDMD and IVODMD. Treating CW with ammonia and/or *T. harzianum* significantly increased (p < 0.0001) ammonia and TVFA concentrations (p < 0.0001). The treatments also significantly increased the digestibility of dry matter (4%–12%), OM (6%−19%), NH_3_ (0.9−10 mM), and TVFA (35−142 mM). Moreover, as the fractions of NDF (p < 0.0392), ADF (p < 0.0046), ADL (p < 0.0001), and cellulose (p < 0.0001) decreased, there was a significant increase in the digestibility of these fractions (Figure-1).

**Table-2:** *In vitro* digestibility and fermentation characteristics after 48 h of incubation.

Items	Treatment				SEM	p-value

	T0	T1	T2	T3		
Dry matter digestibility (%)	42.38^a^	46.82^b^	51.62^c^	54.68^d^	1.10	<0.0001
Organic matter digestibility (%)	44.50^d^	50.82^c^	61.72^b^	64.29^a^	1.86	<0.0001
TVFA (mM)	79.23^c^	114.289^b^	221.84^a^	129.15^b^	12.37	<0.0001
NH_3_ (mM)	4.12^c^	14.56^a^	5.02^c^	10.41^b^	0.99	<0.0001

^a,b,c,d^With divergent superscripts, the means within the similar row differ significantly (p < 0.05), T0=Control, T1=Ammoniated citronella waste, T2=Fermented citronella waste with *T. harzianum*, T3=Ammoniated-fermented (amofer) citronella waste, SEM=Standard error of mean, TVFA=total volatile fatty acid, NH_3_=Ammonia

**Figure-2:**
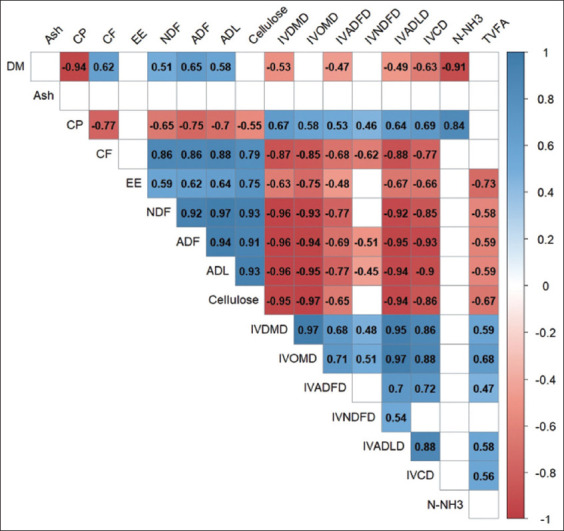
Correlation among the chemical composition, rumen fermentation parameters and *in vitro* digestibility of citronella waste. IVDMD=*In vitro* dry matter digestibility, IVOMD=*In vitro* organic matter digestibility, IVNDFD=*In vitro* neutral detergent fiber digestibility, IVADFD=*In vitro* acid detergent fiber digestibility, IVADLD=*In vitro* acid detergent lignin digestibility, IVCD=*In vitro* cellulose digestibility, TVFA=Total volatile fatty acid.

Correlations among the chemical composition and *in vitro* digestibility of CW are shown in Figure-2. Our findings indicated that the increase in IVDMD and IVOMD of CW was primarily attributed to increased CP content and reduced EE, CF, NDF, ADF, ADL, and cellulose content. Furthermore, the CP content of CW correlated positively with digestibility and levels of N-NH_3_. The EE, NDF, ADF, ADL, and cellulose contents were negatively correlated (p < 0.05) with IVDMD, IVOMD, and TVFA. In the present study, the CF content in CW showed a negative correlation (p < 0.05) with IVDMD and IVODMD. Conversely, there was a positive correlation between IVDMD, IVOMD, IVNDF, IVADF, IVADL, IVCD, and TVFA.

## Discussion

### Chemical composition

Treating CW with ammoniation (T1) yielded the greatest decrease in dry matter, similar to those observed in rice straw [8] and palm press fiber [32]. Similarly, Pan *et al*. [18] and Khasanah *et al*. [33] reported a decrease in their treatment of rice straw and coffee bean husk, respectively, with *T. harzianum*, which is in line with the reduction in DM found in T2 and T3 in the current study. This suggests that the reduction in the dry matter of the treated material is attributed to unavoidable losses associated with increased moisture content (due to the addition of water to production process) and the reaction of ammoniation and/or degradation of fiber constituents by *T. harzianum*. These processes are influenced by the ammonia’s ability to modify the cell wall fraction and the reliance of *T. harzianum* on agricultural by-products for its energy needs.

The protein content of citronella ammoniated, fermented, and amofer increased by 5.3%, 2.3%, and 5.9%, respectively. Furthermore, CF decreases in citronella ammoniated fermented at 2.3%, 2.9%, and 6.6%. Amofer treatment (T3) significantly improved CP by 77% and reduced CF by 20% compared to the control (T0). This study conforms to Datsomor *et al*. [8]. The inclusion of urea as a nitrogen source in ammoniation treatment progressively boosted CP concentration. Ammoniation can stretch the lignocellulose linkage by altering its bonds [34]. A study by Fariani *et al*. [32] explained that ammoniated palm press fiber with 4% urea could increase CP from 3.74% to 6.26% and decrease CF from 47% to 38.39%. Meanwhile, the increased CP of T2 and T3 could result from either colonization or proliferation of the material fermented by the fungi mycelia, fungus capture of available N and extracellular proteinaceous enzymes released by fungi or a combination of all or some of these factors [8]. According to Ahmed *et al*. [35], the protein content of fungal mycelia is quite high, explaining the higher CP levels in T3 compared with T1. In addition, the concentration of extracellular proteins produced by *T. harzianum* NAS110 in substrate TFM containing lemon peel was 73.73 μg/mL [36]. Pan *et al*. [18] showed that fermented rice straw with Trichoderma could increase CP from 6.14% to 7.44%. Similarly, Roba *et al*. [21] recorded an increase in CP and CF in their treatment of rice husk and sugarcane bagasse with Trichoderma.

The EE content of citronella fermented and amofer decreased by 1.05% and 0.59%, respectively. The EE of T2, which is markedly the least among the treatments, is similar to that reported by Amanullah *et al*. [37], who reported decreasing EE in barley straw silage with inoculant lactic acid bacteria. The decrease in EE in the present study contradicts the report of Datsomor *et al*. [8], who reported that treating straw with basidiomycete white-rot *P. ostreatus* fungi led to increased EE because fungi derive nutrients by decomposing and transforming OM into substances, including lipids. Moreover, treating the substrate with Trichoderma led to increased EE, which is consistent with the finding of Khota *et al*. [11] on tropical grass. This variation could be due to differences in the substrate and metabolism of microorganisms in the processed substrate due to the breakdown of lipids by lipase as an energy source. As shown in Table-1, fermentation treatment of CW with *T. harzianum* (T2) produced a higher TDN than the control (T0). However, the value is less than that of ammoniated CW (T1). The highest TDN increase was obtained in the ammonia fermentation treatment with *T. harzianum* (P4). This study is consistent with the rising OM content among CW treatments because amofer could break down structural carbohydrates in high-fiber feed more effectively and the inclusion of urea as a nitrogen source.

Furthermore, Datsomor *et al*. [8] explained a decrease in NDF and ADF in ammoniated and fermented rice straw, which agrees with our study. The findings of this study indicated a decrease in NDF, ADF, lignin, and cellulose of 3.96%, 5.15%, 4.30%, and 2.96%, respectively. This suggests that fermentation is more effective in reducing cell wall constituents. The decreases in NDF, ADF, ADL, and cellulose in fermentation with *T. harzianum* were 8.12%, 7.63%, 7.44%, and 9.41%, respectively. The greater reduction of NDF and ADF in the fermented CW with *T. harzianum* might be related to the release of many enzymes involved in the fermentation process. According to Zhang *et al*. [38], *T. harzianum* in corn stover-pretreated alkali secreted enzymes such as cellulose (Endo-β-1,4-glucanase; Cellobiohydrolase; β-1,4-glucosidase) at a concentration of 5.80% and hemicelluloses (Xylanase; β-xylosidase, arabimofuranosidase) at a concentration of 72.16%. Moreover, Gooruee *et al*. [36] explained that the total cellulase, pectinase, and xylanase enzyme activities in *T. harzianum* NAS110 were 0.78, 0.99, and 0.74 U/ml, respectively, in substrate TFM containing lemon peel. The hydrolysis of lignocellulose requires the synergistic activity of these enzymes. Endoglucanases break β-1,4-glycosidic bonds in amorphous areas of cellulose, releasing reducing and non-reducing chain ends [39]. Exoglucanases remove dimers (cellobiose) from the ends of the cellulose chain and produce disaccharide cellobiose, which is further cleaved into glucose units by the enzyme β-glucosidase [39]. Zhang *et al*. [38] reported that the enzyme EM0925-ALK, produced by *T. harzianum* at a dosage of 1 mg/poteins/g substrate, could hydrolyze cellulose and xylan at 4.65% and 81.85%, respectively, in corn stover pretreated with alkali. Moreover, Zhang *et al*. [40] reported that the enzyme cocktail EM0925-NT produced by *T. harzianum* could simultaneously and completely hydrolyze cellulose in corn stover-pretreated alkali at an enzyme dosage of 10.8 mg protein/g substrate. Meanwhile, ammonia mimics the effect of alkali treatment [34], which in turn disrupts the bond between lignin and cellulose or hemicellulose. According to Kim *et al*. [41], the decrease in lignin content may be attributed to increased lignin solubility, which arises from the breakdown of ether bonds in lignin or the disruption of ether/ester bonds within the lignin-hemicellulose-cellulose polysaccharide complex. These variations could influence the levels of NDF and ADF in CW.

Accordingly, the combination ammoniation-fermentation process in T3 could be more effective in the delignification of CW. The decreases in NDF, ADF, lignin, and cellulose in amofer CW were 9.80%, 10.61%, 9.49%, and 10.47%, respectively. The structure of lignin has been broken as a result of ammonification, resulting in better conditions for degradation of cell wall constituents, allowing Trichoderma to generate energy for growth and enhance the production of extracellular enzymes to facilitate the breakdown of structural carbohydrates present in high-fiber feed. Furthermore, an increase in substrate CP associated with ammonia treatment can meet the requirements of fungal nutrition and growth for free nitrogen amino.

### *In vitro* fermentation characteristics

The amofer treatment of CW led to an approximately 12.30% enhancement in dry matter digestibility. The CF reduction by *T. harzianum* [32] and the urea utilization by rumen microbes [42] explain this. The ammoniated treatment of CW yielded the greatest improvement in dry matter and organic digestibility, according to a study by Elihasridas *et al*. [5]. Gunun *et al*. [14] reported that the digestibility of dry matter in ammoniated and fermented CW was 43.72% and 42.62%, respectively, while the OM digestibility was 42.69% and 41.35% [14]. A greater improvement in IVDMD and IVOMD was observed in fermented *C. nardus* compared to ammoniated *C. nardus* in the present study. The inconsistency can be explained by factors such as varying microorganism inclusion, urea levels, curing times, and preservation methods applied before treatment, such as the chopping process. Several agricultural biomass byproducts, including wheat straw, have been shown to increase IVDMD and IVOMD following fermentation with the inclusion of Trichoderma [43]. Trichoderma activity appears to enhance the availability of energy and nitrogen for rumen microbes, as indicated by reduced NDF, ADF, and ADL levels and increased CP content.

Increased TVFA and N-NH_3_ production in CW in the present study ranged from 79.23−221.84 mM. The optimal production of VFA is to support the maximum rumen microbial protein synthesis ranges from 70−150 mM [44]. These results showed that the process of ammoniation and/or fermentation had significant increases of 35.05%, 142.61%, and 49.92%, respectively. The rise in VFA production is mainly due to an increase in OM content, particularly carbohydrates, which serve as the primary source of VFA. The study by McDonald *et al*. [44] identified acetic, propionic, and butyric acids (VFA), carbon dioxide, and methane as the primary outcomes of rumen carbohydrate digestion. These acids are produced during the digestion of complex carbohydrates (cellulose, pectin, hemicellulose, pentose, sucrose, and fructant) to simple sugars (glucose-1-phosphate, glucose, fructose, and pentoses). The authors also explained that other fatty acids are also produced in the rumen by deamination of amino acids; these include isobutyric acid from valine, valeric acid from proline, 2-methyl butyric acid from isoleucine, and 3-methyl butyric acid from leucine [44].

The NH_3_ concentration in CW-treated T1−T3 ranged from 5.02 to 14.56 mM, which is within the optimal range for rumen microbial growth. Schwab and Broderick [45] explained that ruminal ammonia concentrations ranging from 5 mM to 11 mM are required to maximize microbial N fluxes from the rumen, depending on the diet and fermentation conditions. The CP content in CW was positively correlated with digestibility and N-NH_3_ levels. These findings agree with those of Ma *et al*. [13], who reported a positive correlation between CP content in rice straw and digestibility as well as VFA production. The high concentration of NH_3_ in ammoniated (T1) and amofer CW (T3) is due to the presence of ammonia, which can be a source of nitrogen for microbes. This is consistent with a meta-analysis study by Wahyono *et al*. [46], who revealed that urea supplementation improved rumen fermentation products such as pH ruminal, butyrate, and NH_3_–N concentration.

The treatment of *C. nardus* waste with ammoniation and fermentation using *T. harzianum* (amofer) resulted in the highest digestibility (Figure-1), with an increase of more than 8% in the NDF and ADF fractions and more than 40% in the ADL and cellulose fractions compared with untreated CW (control). This result can be attributed to *T. harzianum* using ammoniated nitrogen for its growth. Tudzynski [47] stated that ammonia is the preferred nitrogen source for fungi because it is essential for fungal growth and the ability to metabolize various nitrogen sources allow fungi to colonize. As the fungal colony population increases, more extracellular enzymes are produced, including cellulase, which in turn can break down more lignocellulose bonds. The increased ADF and NDF digestibility in the ammoniated-fermented (amofer) treatment agreed with Datsomor *et al*. [8].

## Conclusion

The ammoniate-fermented (amofer) with *T. harzianum* treatment demonstrated the most significant improvements in citronella (*C. nardus*) waste. It effectively increased the protein content by 77% and reduced CF, NDF, ADF, ADL, and cellulose by 21%, 14%, 14%, 61%, and 21%, respectively, compared with untreated CW (control). Thus, improvements in nutrient quality of CW resulted in a substantial 29% increase in IVDMD and a 44% increase in IVOMD compared with the control. The study also revealed positive correlations between protein content and digestibility (IVDMD and IVOMD) and N-NH_3_ levels. Conversely, the fractions NDF, ADF, and ADL had negative correlations with digestibility. Further research is required to explore the potential of other microbial agents, such as *Aspergillus* spp. and *Pleurotus* spp., in processing CW for ruminant fodder.

## Authors’ Contributions

DP, SS, IH, BH, and SPG: Conceptualized the study, contributed to the methodology, participated in manuscript preparation, and acquired funding. RA: Data collection, methodology, and manuscript preparation. GET, YY, FF, M, MI, and PCP: Methodology, formal analysis, and drafted the manuscript. All authors have read, reviewed, and approved the final manuscript.
